# Supplementary material for: Insights into the molecular mechanism of yellow cuticle coloration by a chitin-binding carotenoprotein in gregarious locusts
Source: Commun Biol. 2024 Apr 11;7:448. doi: 10.1038/s42003-024-06149-x (PMC11009388; doi:10.1038/s42003-024-06149-x)
Supplement: Supplementary file 2 — Supplementary information [file 42003_2024_6149_MOESM2_ESM.pdf]

Supplementary information

Insights into the molecular mechanism of yellow cuticle coloration by a chitin-binding carotenoprotein in gregarious locusts

Nikita A. Egorkin[1,2], Eva E. Dominnik[1,3], Eugene G. Maksimov[2], Nikolai N. Sluchanko[1*]

[1]A.N. Bach Institute of Biochemistry, Federal Research Centre of Biotechnology of the Russian Academy of Sciences, Moscow 119071, Russia
[2]M.V. Lomonosov Moscow State University, Faculty of Biology, Moscow 119991, Russia
[3]M.V. Lomonosov Moscow State University, Faculty of Chemistry, Moscow 119991, Russia

Email: nikolai.sluchanko@mail.ru

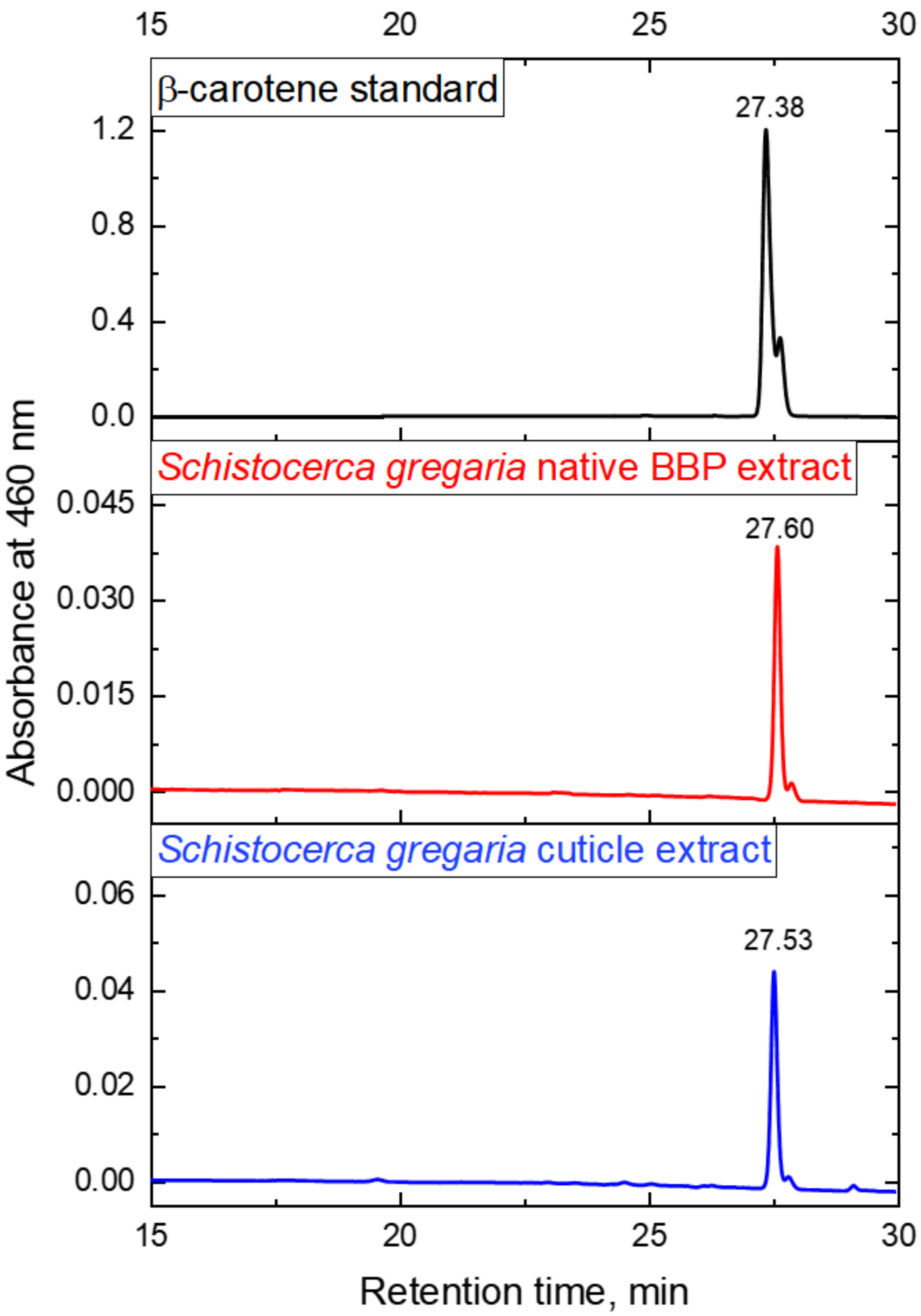

Supplementary Fig. 1. HPLC analysis showing that β-carotene is the predominant carotenoid associated with native BBP purified from the yellow locust cuticles. The upper panel shows β-carotene standard. The middle panel shows the native BBP-associated carotenoid content (the protein was extracted by the standard SEC buffer as described above and then this water-soluble fraction was further extracted by acetone). The bottom panel shows the carotenoid content in the total extract by acetone, demonstrating the essential role of BBP in accumulation of carotenoids.

A **Search parameters**

Enzyme: Trypsin/P: cuts C-term side of KR.
Variable modifications: Deamidated (N), Dehydro (C), Oxidation (M), Propionamide (C)
Mass values searched: 17
Mass values matched: 13

Protein sequence coverage: 89%

Matched peptides shown in ***bold red***.

```
  1 GPHMGVQTCN ASSPDFQLCV RASLQQLIPE LASGVPSIGA EGVDPLRGLP
 51 PIVHNSNGFK VQLDDVSISG LSATLINDVN VDLTSNTIRI QATVPGYITA
101 TGIQTTDAEI MGIPLKGSGP FTISLANPSL AVTLTGAPSA GPNGQTYLRL
151 TSASAAIEPG TPTADIKGFF PQFPPLEAAA SAFASVVAPD VVQSLKPTLD
201 KWLGGVALQR AQAVFSSVSY DALFPGRTPA AVGLYRAVPG LHTLPLPLSA
251 FAYHK
```

B **DNA sequencing result for pET28-His-3C-BBP from T7 forward primer**

GGACGGAACAATTCCATCTAGAATAATTTTGTTTAACTTTAAGAAGGAGATATACC**ATGGGCAGCAGCCATCACCATCATCACCACAG**
CAGCGGCCTGGAGGTGCTGTTCCAAGGTCCTCATATGGGCGTGCAGACTTGTAACGCTTCTTCACCTGACTTTCAGCTGTGTGTTCG
TGCGTCATTACAGCAGTTAATCCCAGAGTTGGCGTCGGGGGTTCCGAGCATTGGTGCTGAGGGTGTTGATCCCTTGCGCGGGTTAC
CGCCTATTGTGCATAACTCCAATGGCTTCAAAGTACAACTTGATGATGTGTCTATTAGCGGTTTGAGCGCCACATTAATCAACGATGT
AAACGTCGACCTTACCTCCAACACCATTCGAATTCAGGCTACAGTTCCCGGGTATATTACTGCGACCGGCATACAGACTACGGACGC
GGAAATTATGGGTATTCCTTTGAAAGGTAGTGGCCCGTTTACCATAAGCCTGGCTAACCCGTCGTTAGCAGTTACCCTCACGGGCGC
CCCGTCCGCGGGGCCTAACGGTCAAACTTATTTGAGACTGACGTCTGCCAGCGCTGCTATAGAGCCGGGCACTCCAACCGCCGATA
TCAAGGGCTTTTTCCACAATTTCCACCTCTTGAAGCAGCAGCATCAGCATTTGCGTCAGTAGTGGCCCCAGACGTGGTACAATCAT
TAAAACCGACTTTAGATAAATGGTTAGGCGGGGTCGCGCTCCAGCGCGCTCAGGCTGTATTTTCGTCAGTTTCTTACGACGCGCTCT
TTCCCGGCCGGACCCCAGCAGCCGTGGGTCTGTATCGTGCGGTTCCGGGGCTCCACACCCTGCCACTTCCTCTGAGTGCTTTCGCAT
ATCATAAGTAGCTCGAGCACCACCACCACCACCACTGAGATCCGGGCTGCTAACAAAGCCCGAAAGGAAGCTGAGTTGGCTGCTGC
CACCGCTGAGCAATAACTAGCATAACCCCTTTGGGGCCTCCTAAACGGTCTTGAGGGGTTTTTTGCCTGAAAGGAGACTATATCCGAT
GGCGATGGGAACGCGCCTGTACCGGCGCAATAGCGCGGCGTGTGATGATAACGCGCGCAGACCGTGCAACCGCATTAGCGAGCTG

TAG – stop codon; **ATG** – start codon. Underlined are *Nco*I (CCATGG), *Nde*I (CATATG) and *Xho*I (CTCGAG) sites.

C

>HisBBP **3C site**
MGSS*HHHHHH*SSG(**LEVLFQG**)PHMGVQTCNASSPDFQLCVRASLQQLIPELASGVPSIGAEGVDPLRGLPPIVHNSNGFKVQLDDVSIS
GLSATLINDVNVDLTSNTIRIQATVPGYITATGIQTTDAEIMGIPLKGSGPFTISLANPSLAVTLTGAPSAGPNGQTYLRLTSASAAIEPGTPTA
DIKGFFPQFPPLEAAASAFASVVAPDVVQSLKPTLDKWLGGVALQRAQAVFSSVSYDALFPGRTPAAVGLYRAVPGLHTLPLPLSAFAYHK

Supplementary Fig. 2. A. BBP identification by matrix-assisted laser desorption/ionization mass-spectrometry on a ultrafleXtreme TOF/TOF instrument (Bruker). B. DNA sequencing result for the BBP plasmid used in this work. C. The translated sequence of the BBP construct used in this work. The His-tag and its cleavage site by human rhinovirus 3C protease are indicated.

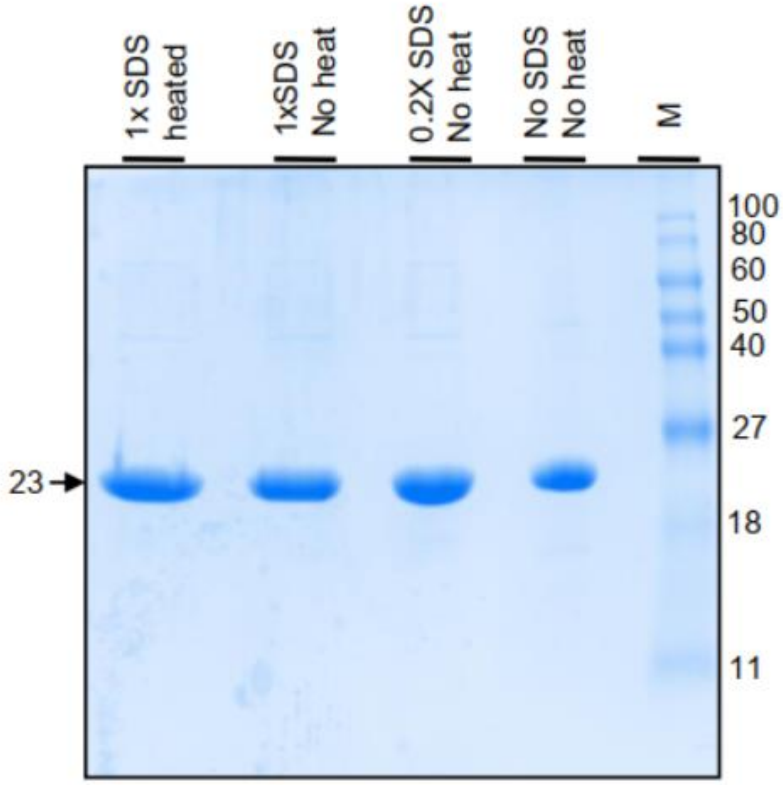

Supplementary Fig. 3. Analysis of BBP by SDS-PAGE with modifications. BBP was loaded either normally prepared with SDS and heat treatment, or was incubated without the latter, at a lower SDS concentration or in its complete absence. “M” designates protein standards with the known M_w values (indicated in kDa). The apparent M_w of BBP (no tag) is shown on the left. Of note, the colored carotenoid zone was dissociated from protein soon after the start of the run.

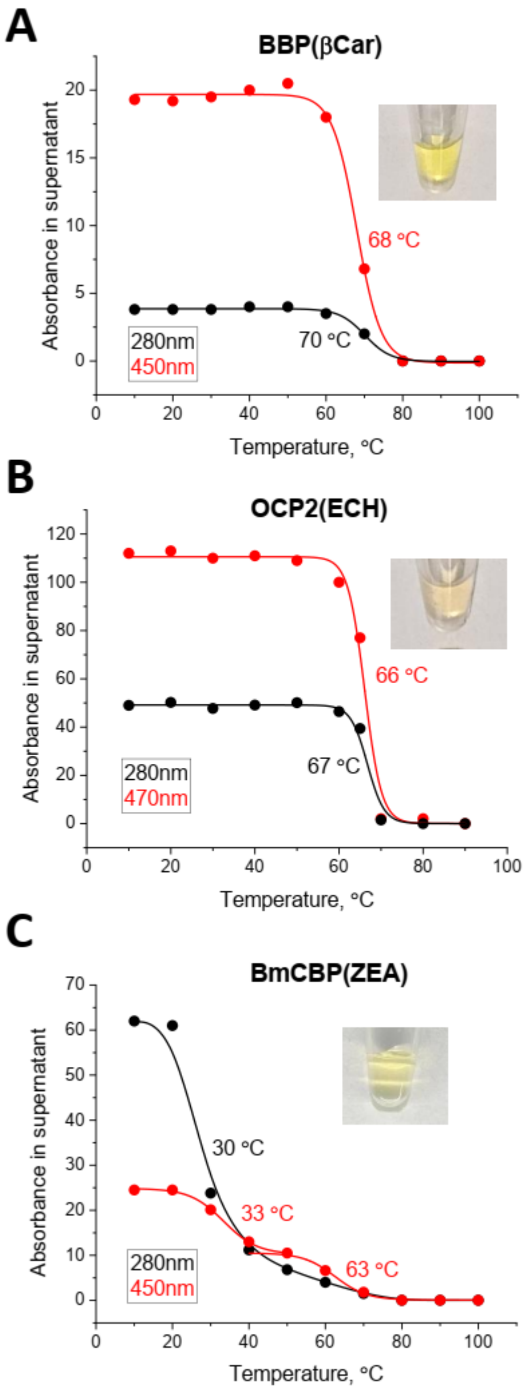

Supplementary Fig. 4. Thermal stability of carotenoproteins BBP (A), OCP2 (B) and BmCBP (C) studied by the SECmelt approach. Aliquots of carotenoproteins were pre-incubated at different temperatures for 20 min, cooled, centrifuged and their soluble fraction after heat treatment was studied by spectrochromatography on a Superdex 200 Increase 5/150 column (Cytiva). Flow rate was 0.45 mL min^{-1}. The amplitude of the chromatographic peak at the native protein position at either 280 nm or visible absorbance wavelength was used to plot the temperature dependences presented. Approximation of the obtained SECmelt curves by Boltzmann equation resulted in half-transition temperatures displayed along with the errors derived from fitting. The appearance of the samples used for the analysis is shown in inserts.

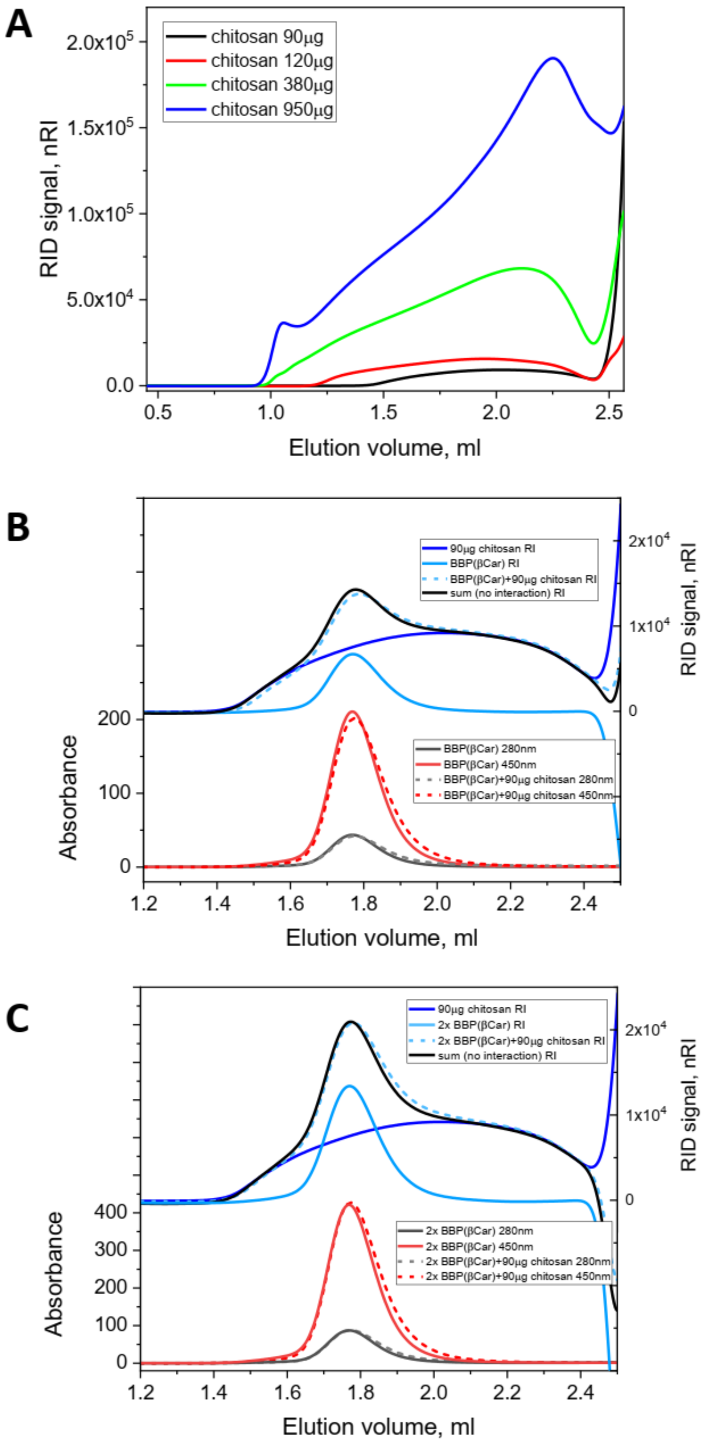

Supplementary Fig. 5. Possibility of BBP interaction with chitosan studied by SEC with absorbance and refractive index detection. A. SEC profiles of four various loads of soluble chitosan on a Superdex 200 Increase 5/150 column (0.45 mL min^{-1}) followed by changes of the refractive index. B,C. The selected chitosan amount was mixed with 1x (B) or 2x (C) concentration of BBP(βCar) and separated by SEC on a Superdex 200 Increase 5/150 column (0.45 mL min^{-1}) followed by changes of the refractive index (upper parts of the profiles) or absorbance (lower part of the profiles). Note that BBP(βCar) is only very slightly shifted (dashed lines) toward the center of chitosan distribution, indicating very poor affinity to chitosan.

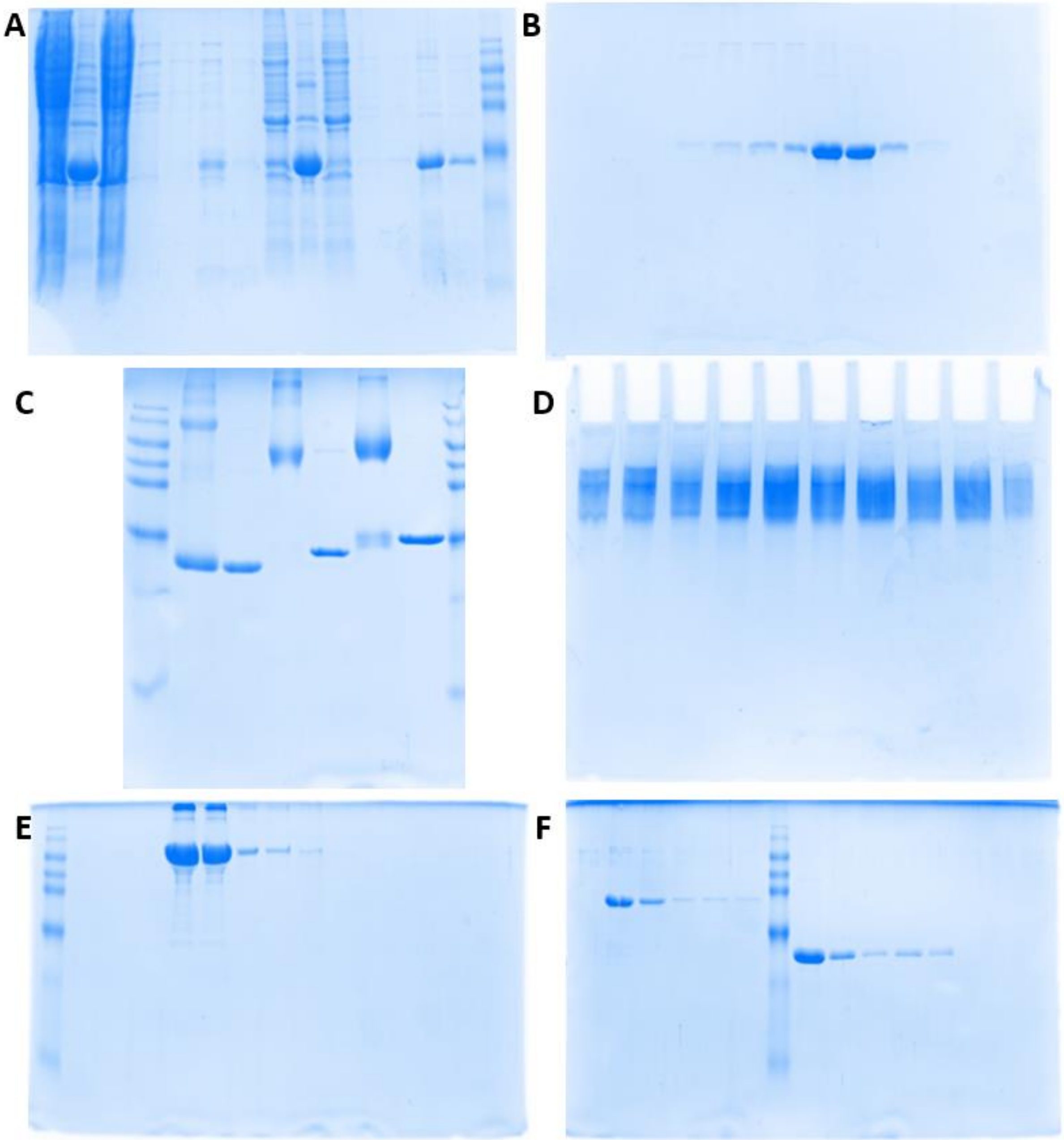

Supplementary Fig. 6. Uncropped gels presented in other figures. **A**. Fig. 1g. **B**. Fig. 1h insert. **C**. Fig. 2d. **D**. Fig. 2e. **E**. Fig. 5c (left). **D**. Fig. 5c (right).
