# Supplementary material for: Insights into the molecular mechanism of yellow cuticle coloration by a chitin-binding carotenoprotein in gregarious locusts
Source: Commun Biol. 2024 Apr 11;7:448. doi: 10.1038/s42003-024-06149-x (PMC11009388; doi:10.1038/s42003-024-06149-x)
Supplement: Supplementary file 3 — Description of Additional Supplementary Files [file 42003_2024_6149_MOESM3_ESM.docx]

**Description of Additional Supplementary Files**

**File name:** Supplementary Data 1
**Description**: The source data behind the graphs in the paper.
